# Table Tennis for Health and Wellbeing: A Rapid Scoping Review

**DOI:** 10.3390/sports14020063

**Published:** 2026-02-05

**Authors:** Louis Moustakas, Kathrin Patzsch

**Affiliations:** Institute for Sport and Sustainable Development, University of Applied Sciences Kufstein, Andreas-Hofer Str. 7, 6330 Kufstein, Austria

**Keywords:** mental health, wellbeing, cognitive skills, motor skills, ping pong

## Abstract

Table tennis has increasingly been adopted as a tool to promote physical and mental health, yet evidence on its outcomes and implementation remains scattered. This study conducted a rapid scoping review to summarise available research on the health outcomes of table tennis within recreational or non-elite settings and identify how table tennis-for-health activities are structured and delivered. Peer-reviewed articles in English were included when they focused the outcomes of table tennis participation on health in community or social settings. Searches across two multidisciplinary databases, complemented by reference screening, led to 17 studies published between 2010 and 2025 being included. Studies were then charted for their methodological, intervention and outcome characteristics. Most studies employed quantitative methods, with experimental or controlled designs predominating, and targeted children, adolescents, older adults, and individuals with conditions such as ADHD or Parkinson’s disease. Across various settings, table tennis was associated with improvements in physical fitness, balance, agility, and body composition, alongside cognitive benefits such as enhanced executive functioning and visual–perceptual skills. Psychological and social outcomes, including improved self-efficacy, emotional regulation, cooperation and social interaction, were also reported. Though no formal quality assessment was conducted, there are clear methodological limitations, including small sample sizes, geographic and gender imbalances, and limited reporting on intervention characteristics that restrict the strength and generalisability of the findings. Overall, this review provides a starting point for trainers and health professionals in the area, presenting promising but preliminary evidence for table tennis as a health-enhancing activity and highlighting the need for more rigorous and comprehensive evaluation.

## 1. Introduction

The promotion of health through physical activity has become an increasing priority for policymakers and service providers over the last decades. Recognising the growing rates of physical inactivity and the consequences of said inactivity, numerous initiatives or approaches have emerged that seek to deploy sport in new or innovative ways that reduce barriers related to cost, time, and performance pressure. Most minors and many adults do not meet minimum physical activity recommendations [1], and many report facing challenges around time, cost and performance pressure when trying to access sport or physical activity offers [2,3]. In turn, the growth in physical inactivity contributes to rising rates of non-communicable disease while making the management of other conditions more challenging [4,5]. Against this backdrop, physical activity formats that can be delivered outside the confines of traditional or competitive sport have become increasingly relevant for public health [6].

One notable and growing approach comes through using table tennis-based interventions to encourage physical activity as well as promote a range of physical or mental health outcomes. Table tennis is seen as a well-known, low-cost and accessible sport that can confer benefits across a range of contexts and target groups, including children, people with neurodegenerative conditions and more [7,8,9,10,11]. Beyond its sporting character, table tennis has increasingly been conceptualised as a structured therapeutic tool, due to its integration of perceptual–motor coordination and cognitive engagement. In line with this, numerous sport organisations have begun developing and deploying table tennis-based approaches within health interventions. These include the French Table Tennis Federation’s PingSanté programme [12], the Ping initiative in the United Kingdom [13], or the PingPongParkinson non-profit in the United States [14]. Globally, the International Table Tennis Federation Foundation (ITTFF) has taken a prominent role in developing and disseminating table tennis approaches to foster social connection, life skill development and health outcomes [15]. Building on this work, the ITTFF are currently working on the development of a handbook and pedagogical resources to support practitioners in applying table tennis to the health field—in activities that move beyond a purely recreational or competitive character in order to intentionally target specific health outcomes. The authors of this paper, along with a fellow consultant, were commissioned to support the development of this handbook and to explore the evidence basis surrounding table tennis for health.

It is in this applied context that this paper finds itself. To ensure that these resources are evidence-based and relevant for practitioners, we have worked to summarise evidence of table tennis’ outcomes across a range of health areas as well as understand the conditions, approach and settings that allow for table tennis interventions to foster those outcomes. As such, we have conducted a rapid scoping review to answer two key, related questions: what physical and mental health outcomes are associated with table tennis within recreational or non-elite settings; and (2) how are table tennis-for-health activities structured and delivered? Beyond its applied focus, this study also builds on existing literature and reviews. Previous work has often been narrative in nature [7,8], focused on neurodegenerative conditions [7,10] or minors [8,16]. For example, Ortega-Zayas and colleagues [16] performed a systematic review looking at table tennis outcomes in primary schools, capturing a small sub-set of seven articles looking at how the sport can be used in the school context, pupils’ motivations to participate and some of its outcomes. In contrast, this review captures both a wider range of outcomes and important intervention characteristics—including duration, format, target group, and pedagogical approach—to provide concrete guidance for implementers, giving it significant practical value.

## 2. Materials and Methods

Our method is based upon the conventional structure of a scoping review, while also making certain concessions for time and logistical purposes. Thus, a rapid evidence analysis (REA) approach was used alongside the general principles of a scoping review to capture the outcomes and conditions associated with the usage of table tennis for health and wellbeing promotions. REA is commonly used in more practical or applied projects and are likewise appropriate for investigating new or emerging topics. Thus, REAs aim to be systematic yet make concessions concerning the breadth, depth, and complexity of the process by limiting certain aspects typically associated with traditional systematic literature reviews [17,18]. Here, we employ the general scoping review approach proposed by Arksey and O’Malley [19] to structure our review, but make some concessions around the types of content and amount of reviewers required to screen content. Ultimately, this has allowed us to produce a still-rigorous summary while also ensuring the timely integration of evidence within the developed handbook and pedagogical materials. The review followed the five steps presented by the authors, namely (1) identifying the research questions; (2) identifying relevant studies; (3) study selection; (4) charting the data; and (5) collating, summarising, and reporting the results. Overall, our review lasted roughly three months between October and December 2025. Given the rapid and applied nature of the work, no protocol was pre-registered and we have not engaged in formal quality assessment of the included literature, but we have otherwise followed the PRISMA extension for scoping reviews [20] and provided the checklist in the Supplementary Materials as Table S1. In the following subsections, we detail the process associated with each of the five steps.

### 2.1. Identification of the Research Questions

According to Arksey and O’Malley [19], research questions should guide the search strategy, not be overly narrow that they limit the analytical process and be broad enough to identify the relevant literature. As such, in line with the aims of our project, we developed two research questions to structure our work: (1) what physical and mental health outcomes are associated with table tennis; and (2) how are table tennis-for-health activities structured and delivered?

### 2.2. Determination of Relevant Studies

Two multidisciplinary, inclusive, and thematically relevant databases—Dimensions and Lens.org—were selected, and various search combinations were piloted. A final search string was chosen that captures a breadth of results while also ensuring that the type of sport and context of interest are appropriately captured. For instance, here, our interest lies in the usage of table tennis in more general or community settings, and we thus excluded terminology related to performance contexts.

Our search string, outlined in Table 1, was then deployed on 23 October 2025, simultaneously through Dimensions and Lens.org. These are two freely accessible databases combining publications, patents, and funding data. Though not curated in the same way as Web of Science or Scopus, they capture many of the traditional sources present on these more selective databases as well as emerging or open access sources [21], including those from countries where table tennis enjoys heightened popularity. Furthermore, given their current status as no-cost resources, they are advantageous for universities and applied organisations that cannot afford access to other more costly databases. The exclusive use of these two databases reflects a deliberate methodological decision aligned with the rapid review design of the study. Additional freely accessible databases, such as PubMed or Google Scholar, were not included primarily due to time constraints and the substantial duplication of records they would introduce, which would have required extensive additional screening with limited expected gains in relevant studies. PubMed, in particular, shows considerable overlap with Dimensions and Lens.org [21], while Google Scholar, despite its breadth, lacks the standardised export functionalities required for optimal scoping review workflows. To ensure comprehensiveness within the time frame of our rapid review, we instead opted to perform a scan of selected references and use an additional digital tool to capture relevant documents, two processes which we describe in the next section.

### 2.3. Study Selection

**Figure 1:**
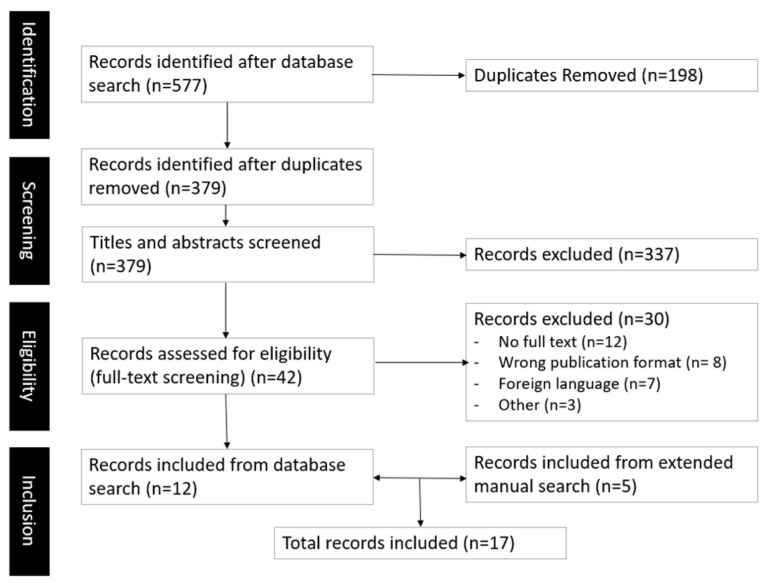
Prisma flow chart for rapid scoping review.

Rayyan software (Cambridge, MA, USA) was used to manage and streamline the process of abstract and full-text screening. Rayyan is a web-based systematic review software that supports rapid screening of the literature through collaboration, blinding, and machine-assisted relevance ranking [22]. In line with the rapid nature of our work, only one author reviewed each abstract and subsequent full text. During the respective screening and eligibility phases, reviewers could consult the abstracts and, later, full texts within the software, and mark it for inclusion, exclusion or as a “maybe” to indicate the need for a collaborative decision. In cases of uncertainty, both authors consulted and came to a unanimous decision aligned with the developed inclusion criteria. In line with this process, no inter-rater reliability was assessed. Full-text inclusion occurred when studies reported on physical health, mental health, or general wellbeing outcomes (e.g., quality of life, inclusion) related to participation in non-performance table tennis activities. Texts discussing table tennis in competitive or performance contexts only were excluded, as were texts discussing non-health related aspects of table tennis initiatives. Furthermore, texts that mentioned table tennis as part of an intervention with other sports but do not isolate for table tennis were similarly excluded. To ensure that relevant sources were not missed, we performed a manual extended search by scanning the reference lists of three narrative or systematic reviews connected to our topic (see [7,9,10]) as well as the results produced by a query submitted to the Elicit search software (Covina, CA, USA). Elicit is an AI-driven academic search platform that organises literature research around explicit research questions, enabling targeted retrieval and analysis. The full inclusion and exclusion criteria are summarised in Table 2, while the overall text selection process is depicted in Figure 1.

### 2.4. Charting the Data

The next stage of the process involved charting and data extraction from the included studies. We conducted this process using a shared spreadsheet and collected bibliographic, methodological, and activity-related information for all included studies. In terms of bibliographic and methodological information, we collected the following: author(s); year; journal; country of study; research questions; study design; and sample size and information. At the activity level, we mapped out the health outcomes measured, the timing and frequency of participation, the setting, the individuals delivering activities, and any pedagogical approaches considered within those activities. Though some items, such as study design or sample size, are straightforward to chart, others require a clear frame to ensure consistent interpretation. Thus, as we detail below, we use specific breakdowns concerning settings, outcomes and the nature of activities. When data could not be categorised, we mark these in the summary extraction sheet, Table 3, with an “x”. The full extraction sheet is provided in the Supplementary Materials as Table S2.

Of note, we categorised the activity setting according to World Health Organisation (WHO) classifications, distinguishing between sport, school, health, workplace and special population settings (see [23]). For health outcomes, we inductively categorised these into four main categories, namely physical (e.g., muscle mass, cardiovascular system, bone density), cognitive (e.g., visual–perceptual functions), psychological (e.g., anxiety, depression, self-efficacy), and social (e.g., interaction with others, sense of belonging) outcomes. The exact outcomes reported were extracted narratively following this initial categorisation.

To better understand the nature and unique features of interventions, we also narratively extracted the table tennis-specific characteristics that are believed to support health outcomes and categorised the nature of the activities delivered as being either cooperative or competitive in nature [24].

The first and second authors undertook a pilot charting process that involved data extraction from two texts to become familiarised with the process and ensure consistency. Thereafter, charting was conducted by the second author and reviewed by the first author. Furthermore, both authors discussed any open questions and uncertainties to ensure continued consistency in the charting process.

**Table 3:** Summary of included studies.

Reference and Year	Method	Setting	Country	Target Group	Sample Size	Sample Size—Table Tennis	Duration/Frequency	Total Time (Hours)	Objectives	Character of the Activity
Aparicio-Chueca & Muñoz-Vila, 2025 [9]	Cross-sectional	Sports setting	Spain	Various groups who regularly practised table tennis; majority over 50 years	329	329	X	X	Physical; cognitive; psychological; social	X
Chen et al., 2024 [25]	Experimental/Controlled	Health setting	Taiwan	Children (with mild intellectual disabilities and borderline intellectual functioning)	91	45	16 weeks, 3 × 60 min per week	48 h	Physical; cognitive	Cooperative
Deprá et al., 2022 [26]	Experimental/Controlled	X	Brazil	Elderly (≥60 years )	16	8	16 weeks, 2 × 75 min per week,	40 h	Physical	X
Han & Wang, 2023 [27]	Experimental/Controlled	School setting	China	Students (grade 7 of a high school)	60	30	9 weeks total, time, and frequency according to the school physical education class	X	Physical	X
Hertting et al., 2020 [28]	Qualitative	Workplace setting	Sweden	Adult employees	12	12	Initial workshop, five table tennis sessions once a week for five weeks, follow up and final workshop	X	Physical, social, emotional	X
Inoue et al., 2021 [29]	Longitudinal/Observational	X	Japan	Patients (with Parkinson’s disease)	12	12	6 month, 1 × 6 h per week	156 h	Physical, cognitive, psychological	Competitive
Liu, 2025 [30]	Cross-sectional	School setting	China	Children (8–14 years)	312	312	Min. 6-month, Training min. 3 times per week	X	Physical, cognitive, psychological	X
Naderi et al., 2021. [31]	Experimental/Controlled	X	Iran	Elderly man (>65 years)	40	20	Min. 6 month, 3–5 × 1.5 h per week	117 h–195 h	Physical	X
Naderi, Degens et al., 2018 [32]	Cross-sectional	X	Iran	Elderly man (68.8 ± 4.6 years)	40	20	No intervention (regular recreational table tennis players—training experience 5–19 years, 2–5 × 1.5–3 h per week	X	Physical	X
Naderi, Zagatto et al., 2018 [33]	Cross-sectional	X	Iran	Elderly men (65—75 years)	40	20	No intervention (regular recreational table tennis players—training experience 5–19 years, 2–5 × 1.5–3 h per week	X	Physical	X
Obadiora & Obadiora, 2018 [34]	Experimental/Controlled	Special population (prison)	Nigeria	Man (20–35 years)	140	14	8 weeks	X	Social	X
Pan et al., 2016 [35]	Experimental/Controlled	X	Taiwan	Children (boys 6–12 years old with ADHD)	32	32	12 weeks, 2 × 70 min per week	28 h	Social; cognitive	Competitive & cooperative
Pan et al., 2019 [36]	Experimental/Controlled	X	Taiwan	Children (boys 7–12 years with ADHD)	60	15	12 weeks, 2 × 70 min per week	28 h	Physical; cognitive	Competitive & cooperative
Pradas et al., 2021 [37]	Cross-sectional	Sports setting	Spain	Children (10–11 years)	374	182	No intervention: Table Tennis players must have maintained a training routine of at least 5 h per week over two years	X	Physical	X
To-aj et al., 2025 [38]	Experimental/Controlled	X	Thailand	Adults (40–70 years)	31	16	30 days, 4 h a day in two sessions	120 h	Physical	Competitive
Wei et al., 2024 [39]	Cross-sectional	Sports setting	China	Elderly (60–70 years)	93	29	No intervention: Table tennis players a required to engage in 40–60 min of exercise at least 3 times per week for a duration of more than two years	X	Cognitive	X
Yu et al., 2025 [40]	Experimental/Controlled	School setting	China	University students (17–21 years)	241	241	4 month, 1 × 90 min per week, (15 sessions in total)	22.5 h	Physical; psychological	X

### 2.5. Collating and Reporting Results

Both study-level frequency analysis and deductive coding were used to summarise and report the results. The variables extracted for the frequency analysis included publication year, country of study, journal, study design, study sample, and activity details. Based on this, both narrative and quantitative summaries were generated around these elements. In line with the rapid nature of the review and to ensure a timely summary of evidence, no formal quality assessments were conducted.

## 3. Results

### 3.1. Study Populations

The total sample size of the studies ranges from 12 to 329, averaging 113 per study. Not all participants took part in table tennis activities; some were part of a control group or took part in other sporting activities for comparison purposes. This was the case in 11 studies, wherein the table tennis-specific sample ranged between 8 and 182 participants. There were fewer than 20 participants that played table tennis in five studies. There were more than 50 participants that played table tennis in four studies, of which all four studies had more than 100 participants. The studies were conducted with children, adolescents, adults, and elderly. Most of the studies were conducted with children and adolescents between the ages of 6 and 21 (n = 7), followed by elderly people over 65 years of age (n = 6). Six of these studies refer exclusively to men. There are no studies that refer exclusively to women. Four studies focus on participants with diagnosed diseases such as Parkinson’s or ADHD or participants with disabilities (intellectual disabilities, borderline intellectual functioning).

### 3.2. Study Methodologies

The selected studies predominantly use quantitative approaches (n = 14), with only one study using a qualitative approach and two using mixed-methods approaches. Within the quantitative studies, experimental or controlled designs were the most common (n = 9), followed by cross-sectional approaches (n = 6). Most studies documented a targeted table tennis intervention, while four studies investigated outcomes associated with general table tennis participation.

In terms of experimental or controlled designs, one example comes from Chen and colleagues [25]. Here, the effect of 16-week table tennis training (TTT) and standard occupational therapy (SOT) on visual perception and executive functions in school-age children with mild intellectual disabilities and borderline intellectual functioning was examined. The children were randomly assigned to SOT or TTT intervention or the control group. The Test of Visual Perceptual Skill—third edition (TVPS-3), Wisconsin Card Sorting Test 64-card version (WCST-64) and the Stroop test were used to assess visual perception and executive functions both before and after the intervention. Another example is the study by Deprá et al. [26]. The study examines the effects of table tennis on physical and functional fitness in elderly people. The participants were divided into a control group and an experimental group. The experimental group underwent table tennis training, while the control group continued their normal everyday activities and were instructed not to start any new sporting activities. Balance and physical fitness were measured both before the start of the 16-week intervention and after the completion of the intervention. To assess the balance, Stabilography analysis was used; to assess physical fitness, the battery of Rikli and Jones and Side Steps Teste of Rodrigues was used.

A number of studies also use cross-sectional approaches. Cross-sectional approaches measure variables at a single point in time to compare groups or examine associations. For example, Aparico-Chueca and Muñoz-Vila [9] measure the perceived physical, cognitive, emotional, and social benefits of regular table tennis practice using a structured questionnaire completed by regularly practising table tennis players. Afterwards, Exploratory Factor Analysis, Regression Analysis, Correlations and Cluster Analysis were used to analyse the data collected at that specific point in time.

### 3.3. Table Tennis Setting

While they all deal with topics related to health and wellbeing, the studies take place in a variety of different settings. These settings include educational institutions (schools and universities), workplaces, prisons, recreational facilities, and therapeutic contexts. Using the aforementioned WHO classifications [23], we note that school (n = 3) and sports (n = 3) settings were the most frequent, whereas individual studies also took place in special populations, workplace and health settings. We could not extract the setting type from eight studies. These studies also took place across nine different countries, with China (n = 4), Taiwan (n = 3), Iran (n = 3), and Spain (n = 2) being the most prominent. Further studies took place in Brazil, Japan, Nigeria, Sweden, and Thailand.

### 3.4. Duration of Table Tennis Activities

No clear trend can be identified in the 11 studies that document the duration or frequency of table tennis activities. The total playing time for the entire study period is 22.5 h to 195 h. The duration of participation ranges from 30 days to 6 months, the playing time per session from 40 min to 6 h, and the number of weekly sessions from 1 to 7.

The playing time is most comparable on a weekly basis. Here, the range extends from 90 min of weekly table tennis activity to 1680 min (=28 h). One study uses 90 min of activity time, five studies use activity times between 120 and 180 min, two studies use between 270 and 450 min, and one study uses 28 h of activity per week. The majority of studies therefore propose an activity time of 2 to 3 h per week.

### 3.5. Health Objectives

The retained studies target a variety of objectives, which we can broadly categorise into physical, social, psychological, and cognitive outcomes. For example, Pradas and colleagues [37] tracked physical outcomes such as bone development or physical fitness, whereas Liu [30] looked at psychological outcomes such as social behaviours and cooperation. Summarising these outcomes into physical, social, psychological, and cognitive, we see that physical objectives are the most common (n = 15), followed by cognitive (n = 8) and then psychological or social (n = 5). These objectives were of course not pursued in isolation, but often in concert, with physical and cognitive objectives co-occurring on five occasions and physical with psychological objectives on two occasions.

### 3.6. Pedagogical/Training Approach

Details on the character of table tennis activities’ overall pedagogical or training approach are not described consistently across the studies. Five studies clearly distinguish whether the activities are competitive (n = 2), cooperative (n = 1) or hybrid (n = 2) in nature. Within these studies, authors note that participants can be motivated by the competitive aspect of table tennis and by the opportunity to play against each other [28,29].

Beyond the competitive or cooperative nature of the activities, as well as the duration and frequency outlined above, few studies describe the underlying characteristics or pedagogical approach. Certain studies, however, at least partially document these points. The study from To-Aj and colleagues [38] highlights that a structured table tennis intervention with a coach achieved better results than playing table tennis. The approach from Chen et al. [25] progressively increased the difficulty of the exercises and used both verbal as well as kinetic feedback to support participants. Finally, Pan et al. [36] highlight how they used a constraints-led approach within their activities. The constraints-led approach means adjusting the task, the environment, or individual roles so that participants naturally adapt their movements and attention. For example, this can imply changing the direction or speed of the ball, modifying the timing between hits, or using different coloured balls within a specific table tennis task to cue different responses.

### 3.7. Outcomes of Table Tennis Activities

For children, studies indicate benefits across the four identified health outcome categories. Importantly, this evidence features both intervention studies examining changes following structured table tennis programmes and associative studies reporting cross-sectional relationships or perceived benefits of regular participation. In terms of physical outcomes, regular table tennis training has been shown to improve upper limb performance, aerobic capacity, vital capacity, and general fitness indicators such as muscle strength and bone development [27,37,40]. Children engaged in table tennis also exhibit favourable changes in cardiorespiratory and motor performance when compared to active peers in other sports, with table tennis players showing greater gains in vital capacity and stronger physical fitness profiles [37,40].

Under cognitive outcomes, intervention-based evidence suggests table tennis training is associated with improvements in visual–perceptual functioning, executive functions, reaction time, postural control, visual–motor integration, and performance on tasks such as the Stroop and WCST [25,30]. Children diagnosed with ADHD demonstrated improvements in agility, strength, and total motor composition scores, reflecting enhanced cognitive–motor integration through the table tennis intervention [35].

With respect to psychological outcomes, associative studies suggest that regular participation in table tennis can enhance self-efficacy, emotional regulation, and cooperation, while also reducing antisocial behaviours and symptoms of depression, though the latter effects are modest [30,40].

Finally, in terms of social outcomes, reported through an observational and qualitative design, table tennis participation appears to facilitate positive peer interaction, cooperative behaviours, and improved social functioning among children [30]. Overall, the evidence suggests multidimensional benefits across physical, cognitive, psychological, and social domains, although sample sizes and methodological designs vary across studies.

Among adults, available studies emphasise psychological and social outcomes, particularly participants’ perceptions of table tennis as a health-enhancing activity. Aparicio-Chueca and Muñoz-Vila [9] report that adults perceive table tennis as promoting physical–cognitive wellbeing as well as emotional–social benefits, indicating perceived gains across multiple outcome domains. In a workplace setting, a single table tennis intervention led to positive individual wellbeing effects, reduced stress, and improved social dynamics within the work environment, illustrating combined psychological and social outcomes [28]. And within a correctional setting, table tennis participation strengthened social interaction, inclusion, and social wellbeing, reflecting meaningful social outcomes in highly constrained contexts [34].

For elderly participants, various study types highlight physical and cognitive outcomes, with consistent evidence for improvements in agility, balance, muscle strength, lean mass, bone density, and lipid profiles [26,31,32,33]. Lower body fat percentages, improved physical performance metrics, and enhanced cardiovascular risk markers have also been observed. Cognitive findings include better reactive motor control, indicating maintenance or enhancement of neuromotor and cognitive–motor processing [39]. These improvements reflect notable benefits in physical functioning and aspects of cognitive control during ageing.

For patients with Parkinson’s disease, small preliminary interventions show that table tennis participation appears to support physical outcomes, with improvements in motor experiences of daily living and motor examination scores within three months [29]. Cognitive and psychological outcomes remained unchanged, suggesting domain-specific effects. In terms of safety—a key consideration for this group—table tennis was found to be well-tolerated, with no major injuries or adverse events, supporting its feasibility as a low-risk physical activity for individuals with Parkinson’s disease [29].

### 3.8. Rationale for Table Tennis Usage

The studies also report or discuss potential advantages associated with table tennis and their connection to the aforementioned outcomes. A general structural advantage highlighted in the literature is that table tennis requires minimal space, can be played in both cooperative and competitive modes, and is accessible across skill levels, allowing beginners and advanced players alike to benefit [28].

It also appears to be a safe option for children, the elderly, and the injured, as it exerts less stress on the joints [9]. In addition, because table tennis involves limited locomotor displacement, it may offer greater safety and stability for older participants [26]. Another advantage of table tennis is that it can be implemented at low cost, for example, in a therapeutic setting in outpatient or community health centres [35]. Its global familiarity further reduces learning barriers and supports uptake across cultural contexts [29].

Due to its intermittent and explosive nature, table tennis forces participants to respond in milliseconds; therefore, essential neuromuscular skills such as agility, reaction time, ballistic strength, and coordination are continually required. This can stimulate cognitive function [37]. Moreover, the intermittent work–rest profile of table tennis resembles high-intensity interval training [31] and may contribute to improvements in physical fitness [37].

Table tennis seems to be an activity merging both physical and cognitive training [36]. For a successful performance in table tennis the player has to process visual information and “consider the time latency necessary to adjust motor commands” [35]. Liu [30] also notes that table tennis, due to its dynamic nature, demands “simultaneous perceptual processing, rapid cognitive appraisal, and social–emotional regulation”. Table tennis is therefore a so-called open-skill exercise requiring fast adaptation to unpredictable changes in the environment [39], which can foster “the emergence of neural plasticity and executive functioning” [25].

### 3.9. Excluded Texts

Finally, we report here on the nature of the texts excluded at the full-text screening phase. Of those texts, most (n = 12) were excluded because a full text could not be located. Indeed, many items, though listed on our selected databases, directed to dead links, and could not be located through alternative search channels (e.g., Google Scholar). Another group of studies were excluded for not corresponding to the desired publication type (n = 8)—peer-reviewed articles—and featured predominantly conference proceedings. A further batch of studies were excluded due to being exclusively in a foreign language (n = 7) such as Ukrainian or Korean. Other studies (n = 3) were excluded for not documenting table tennis activities or for not meeting the thematic inclusion criteria.

## 4. Discussion

Overall, our results show that an emerging body of research suggests that table tennis activities can support physical and mental health. In our results, we see that the strongest and most consistent evidence relates to physical fitness and functional outcomes, with more tentative and preliminary evidence for mental health and cognitive effects. Furthermore, these outcomes can be achieved in a range of diverse contexts such as workplaces, educational institutions, community settings and more. At the physical level, studies show that regular table tennis participation can support improved strength, agility and cardiovascular fitness in children, adult and elderly populations [26,27,31,35]. For example, amongst elderly groups, improvements in agility and balance were observed [26] as well as increased muscle strength, lean mass and higher bone density [31]. These physical outcomes are supported by multiple studies and designs, lending greater confidence to their conclusions.

From a psychological and social perspective, table tennis activities have been connected to improved self-efficacy and social relations [9,28,30]. Even in a prison setting, it was found that table tennis has a social interactional effect and can improve social wellbeing among inmates [34]. However, many of these conclusions are based on single studies or associative designs and should be interpreted accordingly. Cognitively, table tennis can likewise play a supporting role alongside other sports for some mental or neurodegenerative disorders. For example, small pilot intervention studies with children with ADHD report improvements in executive function and cognitive performance following table tennis-based programmes [35] while preliminary evidence from studies with people with Parkinson’s disease shows improvements in motor experiences of daily living and motor examination within three months of starting regular table tennis exercises [29].

Yet, despite these apparently promising outcomes, our review also reveals significant concerns or gaps regarding the quality and precision of the evidence offered. Though we did not perform formalised quality checks, the retained texts do face some methodological issues. The average sample size for table tennis participants is about 78 people, and 11 studies assigned less than 35 people to their respective table tennis groups, raising questions about the generalisability of the results even when interventions are controlled. The high proportion of cross-sectional approaches further limits the strength of results. Further specific studies also raise additional concerns. The series of three studies from Naderi and colleagues [31,32,33] feature the exact same sample sizes and target groups, raising concerns about salami slicing and potential inflation of certain outcomes in our analysis.

The literature would also be enriched by considering a broader diversity of target groups and contexts. Asian, and specifically East Asian, countries make up a vast majority of the retained studies. In contrast, Europe, Africa, and the Americas account for only five studies. This highlights a need to capture how and to what effect table tennis interventions are conducted in other cultural contexts. Future directions should therefore include studies implemented in different countries and across more diverse settings, including health or therapeutic environments, where table tennis is increasingly being used but rarely evaluated. Moreover, more detailed comparisons with other open-skill sports could help clarify the mechanisms unique to table tennis. Likewise, the target groups captured in the data skew towards males. For example, the study on Parkinson’s disease concerns only men, and it would be valuable to investigate how table tennis can support women with similar neurodegenerative conditions. Overall, this finding further confirms the presence of a “gender data gap” in sport and health research (cf. [41]) and points to a need to actively encompass gender-diverse target groups within this research area.

The latter points also speak to a broader issue within the retained studies. Information about setting, delivery and intervention characteristics is sorely missing. Clear information about the setting, duration of activities, the persons delivering activities, the training methods or the setup or characteristics of activities is consistently absent across the studies. Indeed, not a single study reports on all these aspects. This highlights a pressing need to better document and understand what works, for whom, and under what circumstances when it comes to table tennis for health. Future work must consider the exact activity or intervention characteristics, such as frequency, duration, pedagogy, and instructional strategies, which lead to particular outcomes. Relatedly, there is little indication of whether and how activities are adapted to target groups, and this limits our ability to understand whether table tennis’ effects depend on population-specific tailoring. In addition, the role of motivation and participants’ subjective perceptions of enjoyment and meaningfulness remains largely unexplored, despite these factors being well-established mediators of sport-based health outcomes. Though more managerial-focused work exists already [13], future in-depth and qualitative research would do well to explore how practitioners can optimise the setting, delivery, and pedagogy of table tennis-for-health programming. Indeed, other reviews have likewise noted a lack of research on the implementation of table tennis for health [16]. More comprehensive reporting and systematic evaluation practices are essential if the field is to advance beyond preliminary evidence and develop robust interventions that are accepted as not only a recreational, but as a health or therapeutic approach.

The retained studies and their synthesis also outline some important, albeit tentative, practical implications. The reviewed studies suggest that table tennis interventions are most likely to support health outcomes when delivered in a structured, coach-supervised setting as opposed to being simply ad hoc or self-directed activities. Across interventions, positive outcomes are most reported in programmes lasting several weeks to a few months, typically involving two to three sessions per week and a total activity volume of approximately two to three hours weekly. Both competitive and non-competitive formats are represented in the literature, although cooperative or hybrid approaches may be particularly appropriate in health-focused contexts where safety, inclusion, and sustained engagement are often prioritised above performance.

Obviously, our analysis is far from perfect. As a rapid scoping review, this study involved methodological trade-offs that led to several limitations, including in terms of the breadth of results included and the depth of the analysis. Though we suspect we have captured a fairly comprehensive swath of the English-language peer-reviewed literature, our database strategy and time constraints likely resulted in the exclusion of numerous conference, book, and grey literature contributions through the restrictions imposed here. Future work would do well to consider capturing a wider array of source types, including proceedings, books, and reports. Likewise, our reliance on only two databases (Dimensions and Lens.org) was a conscious methodological choice aligned with the rapid nature of the review, but it also constitutes a clear limitation. While these platforms offer broad multidisciplinary coverage, additional databases could have yielded further relevant studies. Accordingly, future reviews would benefit from including both curated and freely accessible databases, such as PubMed, Web of Science, SciLit, or comparable resources, to enhance coverage. Furthermore, our analysis of outcomes is purely descriptive and exploratory, consistent with the aims of a scoping review. Future work could consider applying formal quality appraisal procedures or meta-analytic techniques where appropriate.

## 5. Conclusions

Based on the results of a rapid scoping review of 17 articles, the emerging evidence base indicates that table tennis can be considered as a feasible and accessible activity to support health and wellbeing across a range of non-elite or recreational settings, although the field remains in an early stage of development. Evidence is most robust for physical and functional outcomes, whereas conclusions regarding cognitive, psychological, and social benefits should be regarded as exploratory due to limited studies and sample sizes.

Strengthening methodological rigour, expanding the diversity of study contexts and populations, and improving reporting on intervention design will be crucial steps in ensuring a clearer understanding of how table tennis can be effectively deployed to support health and wellbeing. Future research would benefit from larger and better-controlled intervention studies that more clearly isolate the effects of table tennis, alongside more consistent documentation of key intervention characteristics such as duration, frequency, pedagogical approach, and delivery setting. Expanding research beyond a limited number of countries and predominantly male samples remains essential to ensure that such activities are relevant and effective across diverse groups. Together, such efforts would allow the field to move beyond promising but preliminary evidence towards more robust and practice-relevant knowledge of table tennis for health.

## Figures and Tables

**Table 1:** Overview of search terms and databases.

Boolean Search Terms	“table tennis” AND (“health” OR “well-being” OR “wellbeing” OR “social inclusion” OR “social cohesion”) NOT (“injury” OR “elite”)
Search Area	Title, Abstract, Key Word
Databases	Dimensions Lens.org

**Table 2:** Summary of REA inclusion and exclusion criteria.

	Inclusion	Exclusion
Topic	Articles focusing on the outcomes of table tennis participation on health, wellbeing, or social inclusion of participants in community, health, school, social or refugee settings.	Articles within elite or performance contexts. Articles focusing on injuries or injury prevention. Articles focusing on non-health related aspects of table tennis activities (e.g., management of programmes). Articles focusing on other sports.
Population/Target Group	Players/participants of all ages, and backgrounds	Referees, coaches, or administrators.
Design/Form	Empirical studies using quantitative and/or qualitative methods	Meta-analyses Systematic Reviews Literature Reviews Conceptual or theoretical papers Position papers or editorials
Publication Type	Peer-reviewed journal articles	Grey Literature Theses Books Chapters Preprints
Language	English	Documents not in English
Geographic Scope	Worldwide	None
Timeframe	2010–2025	Documents outside of defined range.

## Data Availability

No new data were created or analyzed in this study. Data sharing is not applicable to this article.

## Supplementary Materials

The following supporting information can be downloaded at https://www.mdpi.com/article/10.3390/sports14020063/s1, Table S1: Checklist from PRISMA extension for scoping reviews; Table S2: Full data extraction sheet.
