# Supplementary material for: Table Tennis for Health and Wellbeing: A Rapid Scoping Review
Source: Sports (Basel). 2026 Feb 5;14(2):63. doi: 10.3390/sports14020063 (PMC12944479; doi:10.3390/sports14020063)
Supplement: Supplementary file 1 [file sports-14-00063-s001.zip › sports-4086935-supplementary Table S2.pdf]

Reference	Paradigm	Method	Setting	Country	Target Group (TT)	Sample Size	Sample Size Table Tennis Group	Duration and Frequency	Total Time (Weeks*Frequency*Session Time)	Persons delivering activities	Primary Objectives	improving or maintaining health?	Pedagogical/training methods	Character of the activity
Aparicio-Chueca, Pilar; Munoz-Vila, Noa	Mixed Method	Cross-sectional	sports setting	Spain	different genders and age groups who regularly practiced table tennis; majority over 50 years	329	329	x	x	x	physical; cognitive; psychological; social	Improving	x	x
Chen, Ming-De; Tsai, Hsien-Yu; Wang, Chih-Chung; Wuang, Yee-Pay	Quantitative	Experimental / Controlled	health setting	Taiwan	**children** (with mild intellectual disabilities and borderline intellectual functioning)	91	45	16 weeks, 3x 60 min per week	48h	coaches	physical; cognitive	improving	increasing difficulty, Verbal and kinetic feedback	cooperative
Deprá, Pedro Paulo; Lopes, Wendell Arthur; Oliveira, Gustavo Henrique de; Costa, Carla Eloise; Da Onório Silva, Vanessa	Quantitative	Experimental / Controlled	x	Brazil	**elderly** (>=60 years)	16	8	16 weeks, 2x 75 min per week,	40h	x	physical	improving	x	x
Han, Liang; Wang, Rui	Quantitative	Experiemntal/Controlled	school setting	China	**students** (grade 7 of a high school)	60	30	time and frequency according to the school physical education class - experiment lasted 9 weeks	x	teachers	physical	improving	x	x
Hertting, Krister; Holmquist, Mats; Parker, James	Qualitative	Qualitative	workplace setting	Sweden	**employees**	12	12	initial workshop, five table tennis sessions once a week for five weeks, follow up and final workshop	x	coaches	physical, social, emotional	improving	physical active group was motivated by competition	x
Inoue, Kenichi; Fujioka, Shinsuke; Nagaki, Koichi; Suenaga, Midori; Kimura, Kazuki; Yonekura, Yukiko; Yamaguchi, Yoshiki; Kitano, Kosuke; Imamura, Ritsuko; Uehara, Yoshinari; Kikuchi, Hitoshi; Matsunaga, Yoichi; Tsuboi, Yoshio	Quantitative	Longitudinal / Observational	x	Japan	**patients** (with parkinson's disease)	12	12	6 month, 1x 6 hour per week	156h	university students	physical, cognitive, psychological	improving	Playing as a match can produce a positve effect in terms of reward processing (maintaining patients motivation)	competitive
Liu, Jiang	Mixed Method	Cross-sectional	school setting	China	**children** (8-14 years)	312	312	min. 6 month, Training min. 3 times per week	x	coaches	physical, cognitive, psychological	improving	x	x
Naderi, A.; Goli, S.; Shephard, R.J.; Degens, H.	Quantitative	Experimental / Controlled	x	Iran	**elderly** man (>65 years)	40	20	min. 6 month, 3 - 5x 1,5hours per week	117h - 195h	instructor	physical	improving	x	x
Naderi, Aynollah; Degens, Hans; Rezvani, Mohammad Hossein; Shaabani, Fatemeh	Quantitative	Cross-sectional	x	Iran	**elderly** man (68.8±4.6 years)	40	20	No intervention (regulary recreational table tennis players - training experience 5-19 years, 2-5x 1,5-3 hours per week	x	x	physical	improving	x	x
Naderi, Aynollah; Zagatto, Alessandro M.; Akbari, Fatemeh; Sakinepoor, Ainollah	Quantitative	Cross-sectional	x	Iran	**elderly** man (65 - 75 years)	40	20	No intervention (regulary recreational table tennis players - training experience 5-19 years, 2 - 5 x 1,5-3 hours per week	x	x	physical	improving	x	x
Obadiora, Ayobami Honestus; Obadiora Adebayo Joseph	Quantitative	Experimental / Controlled	special population (prison)	Nigeria	**man** (20-35years)	140	14	8 weeks	x	x	social	improving	x	x
Pan, Chien-Yu; Chu, Chia-Hua; Tsai, Chia-Liang; Lo; Shen-Yu; Cheng, Yun-Wen; Liu, Yu_Jen	Quantitative	Experimental / Controlled	x	Taiwan	**children** (boys 6-12 years old with adhd)	32	32	12 weeks, 2x 70min per week	28h	coach & research assistans	social; cognitive	improving	motor skills practice and executive function training, were based on a constraints-led approach, dynamic interactions between the individual performer, the environment (peers and coaches) and the task. Coach as a guide and monitorer that facilitated the leraning pocess by manipulating the task constraints and constentley providing verbal and physical feedback , instructor -to-participant ratio 1:2/1:1	competitive & cooperative
Pan, Chien-Yu; Tsai, Chia-Liang; Chu, Chia-Hua; Sung, Ming-Chih; Huang, Chu-Yang; Ma, Wei-Ya	Quantitative	Experimental / Controlled	x	Taiwan	**children** (boys7-12 years with ADHD),	60	15	12 weeks, 2x 70min per week	28h	coach & research assistar	physical; cognitive	improving	x	competitive & cooperative
Pradas, Francisco; ara, Ignacio; Toro, Víctor; Courel-Ibánez, Javier	Quantitative	Cross-sectional	sports setting	Spain	**children** (10-11 years)	374	182	No intervention: Table Tennis players must have maintained a training routine of at least 5h per week over two years	x	x	physical	improving	x	x
To-aj, Oam; Bumrungpanictarworn	Quantitative	Experimental / Controlled	x	Thailand	**adults** (40-70 years)	31	16	30 days, 4h a day in two sessions	120h	coaches	physical	improving	Experimental group: coach and structured programm. Control group: No feedback or structured coaching (Focused on social interaction and enjoyment rather than professional setting	competitive
Wei, Jing-Ning; Zhang, Ming-Kai; Wang, Zhen; Liu, Yu; Zhang, Jian	Quantitative	Cross-sectional	sports setting	China	**elderly** (60 - 70 years)	93	29	No intervention: Table tennis players a required to engage in 40-60 min of exercise at least 3 times per week for a duration of more than two years	x	x	cognitive	improving	x	x
Yu, Dongshou; Shimura, Masako; Kawanishi, Masashi	Quantitative	Experimental / Controlled	school setting	China	**university students** (17-21 years)	241	241	4 month, 1x 90min per week, (15 sessions in total)	22,5h	x	physical; psychological	improving	x	x

Guidelines	
Context	WHO benennt folgende Sektoren: **Sports settings, Health settings, School settings, Workplace settings, Urban planing, Special populations, Raising awareness.** Definition der Sektoren (S.8. ff.): https://iris.who.int/items/c8485d07-3708-4969-bb54-bc9a8998eedc
Primary Objectives	Inductively deduced: **physical** (focuses on physical fitness, e.g., muscle mass, cardiovascular system, bone density), **cognitive** (focuses on cognitive processes, e.g., visual-perceptual functions), **psychologica**l (focuses on emotional aspects, e.g., anxiety, depression, self-efficacy), **social** (focuses on aspects of social interaction, e.g., interaction with others, sense of belonging)
Character of the activity	The structures of well-known sports games are characterized by a combination of certain game characteristics: with or against each other, team or individual; with or without a game object; physical contact, target area, separation of the teams by an obstacle (Bietz & Böcker, 2008, p. 98). Core idea of competing against each other: competitive spirit; Core idea of together: Together (against each other or the game)
Paradigm	Cross-sectional study: A study that measures variables at a single point in time to compare groups or examine associations, without tracking changes over time.<br><br>Longitudinal / Observational study: A study that follows participants over a period of time to observe changes or outcomes, without assigning them to controlled intervention or comparison groups.<br><br>Experimental / Controlled study: A study in which researchers implement a specific intervention and measure its effects, often using pre–post testing and comparison or control groups to assess causality.
